# Robotic Arm-Assisted Total Knee Arthroplasty in the Setting of Combined Extra-articular Deformities of the Femur and Tibia

**DOI:** 10.1155/2020/5489646

**Published:** 2020-07-04

**Authors:** Sharma Cook-Richardson, Rasesh Desai

**Affiliations:** ^1^University of Kentucky Health Care Orthopaedic Surgery & Sports Medicine, Lexington, KY, USA; ^2^Med Center Health Orthopaedics and Sports Medicine, The Medical Center, Bowling Green, KY, USA

## Abstract

In this case, we will describe a 68-year-old man with combined femoral and tibial bone deformities who underwent robotic arm-assisted total knee arthroplasty (RATKA) to treat his severe osteoarthritis in the setting of extra-articular deformities that altered the native anatomical axis and the kinematics of the deformed extra-articular bony structures which chronically generated a neomechanical axis. The combination of severe osteoarthritis with extra-articular deformities made the RATKA method the best surgical treatment option taking into account altered kinematics of the native joint which conventional jig-based total knee arthroplasty would not have prioritized during bony cuts and implant positioning. The patient underwent successful knee arthroplasty with robotic arm-assisted technology with restoration of the mechanical axis.

## 1. Introduction

Robotic arm-assisted total knee arthroplasty (RATKA) uses preoperative 3-D helical computed tomography images of the patient's anatomical bony structure to virtually plan femoral and tibial replacement components, implant positioning, and intra-articular balancing. RATKA's virtual planning may aid in greater component precision, implant accuracy, soft tissue protection, increased patient satisfaction, fewer instrumentation trays required intraoperatively, and overall improved safety of total knee arthroplasty [1]. There have been numerous studies published that compared RATKA methods for the treatment of osteoarthritis in angular deformities and extra-articular deformities to the conventional jig-based total knee arthroplasty (JTKA). Traditionally, the conventional JTKA methods have based the implant replacement components and positioning on the anatomical axis where bony cuts are based on standardized jig placements that have the best fit. However, any changes in the anatomical axis due to structural deformities are not prioritized in the conventional jig-based replacement planning. The computer-assisted surgery such as navigation and patient-specific guide for knee replacement has been used for accurate implant positioning, but these methods do not provide any information on ligament balancing unlike RATKA. The superiority of RATKA in comparison with other techniques is primarily its ability to monitor joint balance and make necessary adjustments intraoperatively to achieve a well-balanced and well-aligned knee. The purpose of this case report is to start the conversation of the best arthroplasty surgical treatment method for deformed bony structures leading to distinct alterations in the anatomical axis of the native joint. Currently, the data is lacking on any benefit of using the RATKA method when compared to the conventional JTKA method to treat osteoarthritis in patients with anatomical axis deformities. In this case, we will describe a 68-year-old man with combined femoral and tibial bone deformities who underwent robotic arm-assisted total knee arthroplasty (RATKA) to treat his severe osteoarthritis in the setting of extra-articular deformities in the femur as well as tibia that altered his native anatomical axis and predisposed changes in the kinematics of his native joint which, with time, chronically generated a new mechanical axis.

Total knee arthroplasty (TKA) has been a longstanding treatment for severe osteoarthritis that may debilitate and/or compromise a patient's functional status. Traditional arthroplasty replacements involved the use of standardized predesigned jigs intraoperatively to position the implant component, and the positioning of the implant was primarily based on the anatomical axis of the joint. Severe osteoarthritis can lead to abnormalities in the anatomical axis due to the development of osteophytes and sclerotic bony surfaces, and these alterations change the kinematics of the native joint. In order to ensure a balanced joint during an arthroplasty procedure, the kinematics of the joint must be taken into account with an understanding of the current mechanical axis. Robotic arm-assisted TKA allows the surgeon to restore normal kinematics to the knee by reproducing alignment, balancing surrounding soft tissue, and decreasing the variability by increasing surgical precision which in turn all together leads to the restoration of the joint line [2]. Also, there was a short learning curve to achieve similar operative times in surgeons who were trained to operate with both systems in which operating time increased by less than 30 minutes [3].

Worldwide use of robotics in orthopaedic arthroplasty procedures has been utilized since the 1980s, and then, the discussion of robots in the future potentially reducing human error by obtaining surgical accuracy, quality control reproducibility, and eliminating excessive outcome variations were the aim for the future as technology continued to improve [4]. To date, there are several studies that have compared the robotic arm-assisted TKA (RATKA) to the conventional jig-based total knee arthroplasty (JTKA) and found the RATKA method to be superior based on the improved accuracy of implant positioning leading to reduced angular outliers (<3 degrees varus/valgus) which can protect the longevity of the implants [5]. Kayani et al. completed a prospective cohort that compared 40 JTKA to 40 RATKA cases all performed by the same surgeon and reported improved early function with the RATKA method based on reduced postoperative pain, decreased requirements of postoperative opiate pain medications, improved straight leg raise and maximum knee flexion at discharge, and shorter length of stay days at the hospital [6]. In a separate study which investigated the patient-reported satisfaction after undergoing either RATKA or JTKA at the 3-month postoperative follow-up, it was concluded that there were larger improvements in walking, standing, and pain with movement with RATKA-treated patients compared to conventional JTKA. This study concluded that the technological advantages that preoperative CT-guided planning and intraoperative real-time haptic feedback increased precision and accuracy of bony cuts and implant component alignment were attributed to the improvement in patient satisfaction and functional outcomes [7].

## 2. Case Presentation

**Figure 1:**
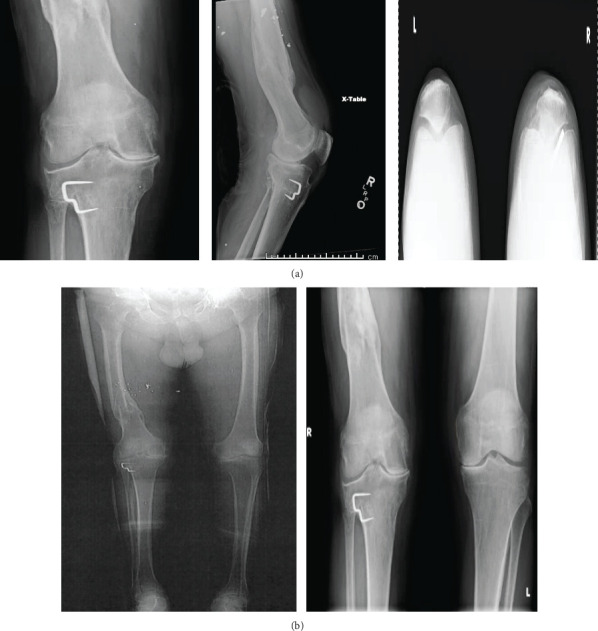
(a) Preoperative anteroposterior, lateral, and sunrise X-ray views displaying tricompartmental osteoarthritis with femoral and tibial deformities, valgus angulation, and tibial osteotomy screws. (b) Preoperative anteroposterior standing bilateral lower extremity X-rays displaying long leg alignment and the mechanical axis with femoral and tibial deformities, valgus angulation, and tibial osteotomy screws.

A 68-year-old man with a history of a distal right femoral diaphyseal fracture that resulted in malunion and right varus knee deformity previously treated with a high tibial osteotomy (HTO) presented with severe osteoarthritic pain of the right knee. The patient had a distal femur fracture at the age of 18 years and was treated conservatively which resulted in malunion and shortening of the leg. At the age of 44 years, he was diagnosed with osteoarthritis and underwent a high tibial osteotomy. However, knee pain continued to get progressively worse especially for the last few years. The patient's social history included using an assisted walking device, being an active cigarette smoker, and combating chronic hypertension. Clinical evaluation and radiographic imaging confirmed the diagnosis of severe bone-on-bone tricompartmental osteoarthritis (Figure 1). The patient's knee range of motion was significantly restricted from full extension to further flexion of only 90 degrees. The patient previously failed various conventional nonsurgical treatments and wanted to proceed with TKA. Given the complexity of this case, the RATKA MAKO-Stryker surgical arthroplasty system was advised as the surgical method in order to plan for implants that would better fit this patient's specific geometric bony anatomy with the plans of bringing the mechanical axis within 3 degrees of neutral biomechanical alignment. A thorough discussion of the risks and benefits of the surgical procedure and preoperative 3-D computed tomographic images of his knee was performed.

### 2.1. Operative Details

The surgical planning included obtaining a MAKO protocol CT scan. The robotic software converts CT images to a virtual model where a surgeon can plan on implant size, positioning, and joint alignment. As demonstrated from images, this patient had a distal femur fracture, which had resulted in malunion. Because of malalignment, the patient had recurvatum deformity. The use of the anatomical axis and jig-based technique would have resulted in the positioning of the knee implant into hyperextension. Also, it would have been difficult to use the jig-based technique in the setting of severe deformity of the distal femur. Also, deformity in the proximal tibia due to a previous high tibial osteotomy would have resulted in difficulties in establishing the mechanical axis. Because of robotic software, we were able to plan the implant position in a way that it would restore the mechanical axis.

After exposure, femoral and tibial trackers were applied. The hip center of rotation was registered followed by medial and lateral malleolus registration. Bony landmark registration was done. Now, we proceeded with removing all the visible osteophytes and remaining ACL. Often times, it is difficult to remove the PCL completely without making the tibial cut in tight knees with longstanding deformities.

**Figure 2:**
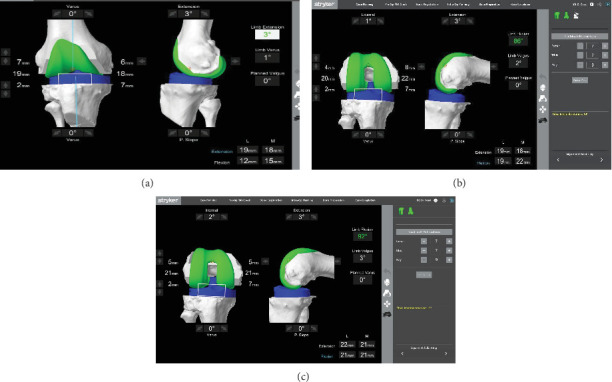
(a) Significant ligament imbalance was demonstrated between flexion and extension even after soft tissue release and removal of ACL and osteophytes. (b) The numbers in the right lower corner demonstrate ligament balance in knee flexion after making the tibial cut and moving the femoral component anteriorly and before rotating the femoral component internally. (c) The final ligament balance was demonstrated in knee flexion and extension after femoral component ration with implants.

We next proceeded with identifying the existing deformity without any correction and with corrective maneuver and checking the balances on the medial and lateral side (Figure 2(a)). With the planned tibial cut of 7 mm from the highest point of the medial tibial condyle with 0-degree varus, we were looking at an extension gap balance of 19 mm laterally and 18 mm medially. This was enough to accommodate an at least 9 mm poly liner. However, in flexion, this gap was 12 mm and 15 mm on the lateral and medial side, respectively. But often, making a cut on the tibia removes the tight PCL and opens up the flexion gap. Also, we all know that tibial cut symmetrically affects the flexion and extension gap. So, we decided to proceed with this tibial cut first.

Next, appropriate tibial cut was made. The previous existing hardware was removed. With the help of the lamina spreader, the balance of the ligament was checked again in extension and flexion. We were able to open up the flexion gap more. Moving the planned femur implant anteriorly and thereby planning to remove more bone posteriorly, we were able to get the numbers closer to the extension gap as evidenced by Figure 2(b).

Now, as evidenced by this figure, on the right bottom corner, the extension gap is 19 and 18 mm and the flexion gap is 19 mm laterally and 22 mm medially. At this point of time, the femoral implant is in 1° external rotation. So now, we needed to close down the medial gap in flexion. We were able to do this by pinning the femoral implant laterally and internally rotating the femoral component. By this maneuver, we were able to predict almost equal gaps in both flexion and extension. We proceeded with the femoral cuts. Trial implants were positioned.

### 2.2. Postoperative Course

**Figure 3:**
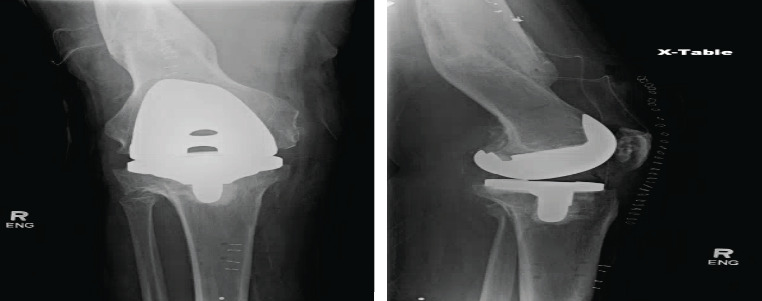
Postoperative anteroposterior and lateral X-ray views of the status of postrobotic arm-assisted total knee arthroplasty and removal of high tibial osteotomy screws.

The patient remained in the hospital for 2 days to ensure that adequate pain management was obtained prior to being discharged home. He returned for a follow-up visit in 3 weeks postoperatively (Figure 3). During this visit, his range of motion was recorded as 0° to 95° flexion, with stable varus/valgus stress testing, negative anterior/posterior drawer signs, palpable distal pulses, and great strength within the quadriceps muscle group. At this point, the patient's knee replacement was deemed clinically stable and the patient was advised to return for a 2-month follow-up.

## 3. Results: Six-Month Follow-Up

**Figure 4:**
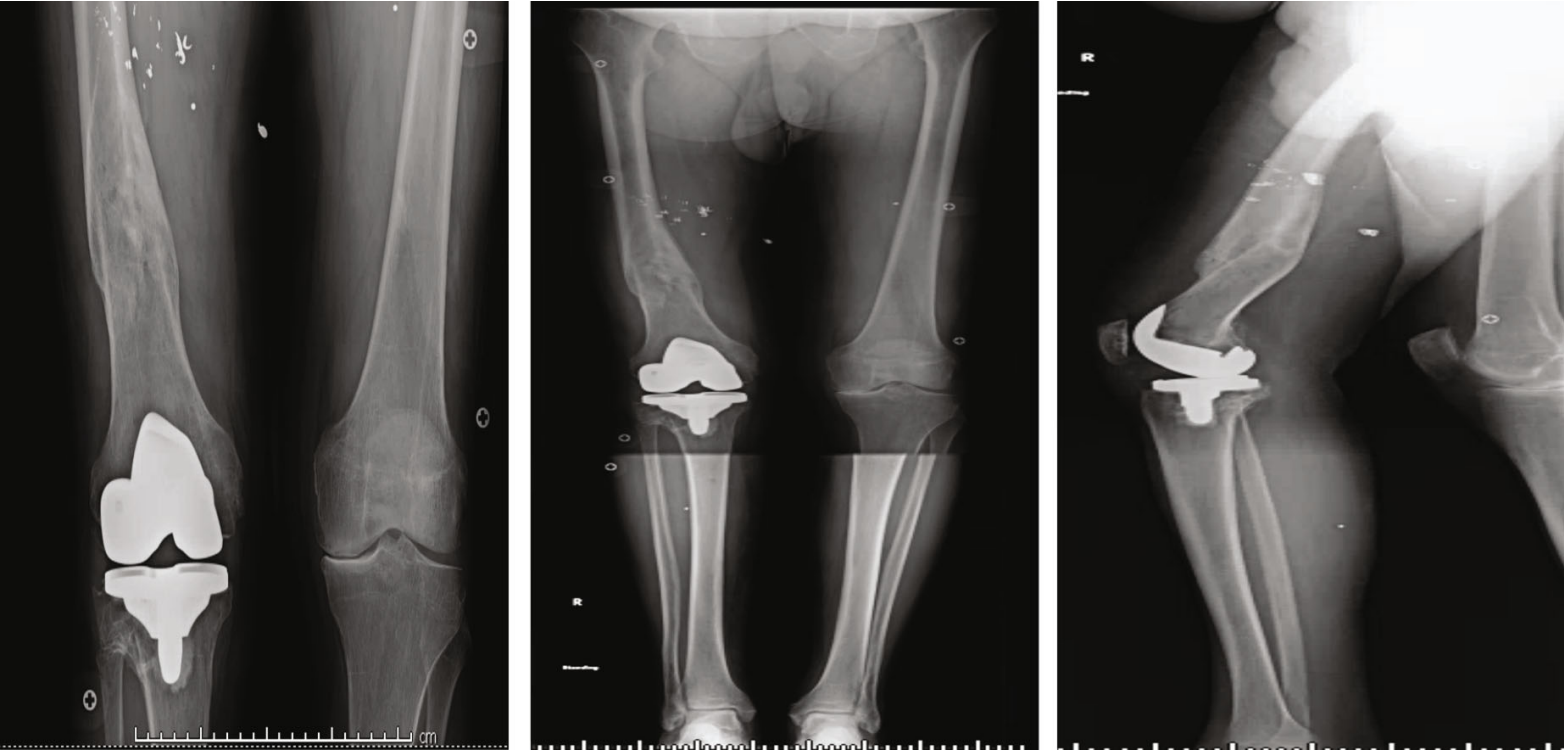
Six-month postoperative X-rays of anteroposterior bilateral knees, bilateral leg length, and lateral leg length flexion.

**Figure 5:**
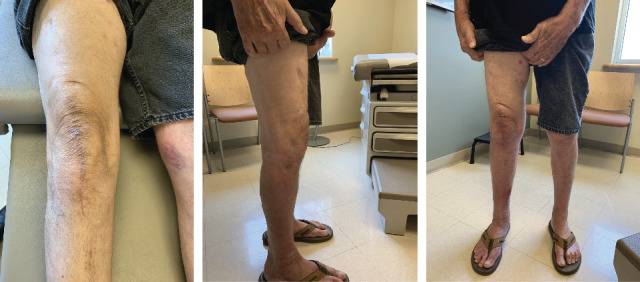
Office photos of frontal, lateral, and close-range postoperative bilateral legs at a six-month follow-up.

**Figure 6:**
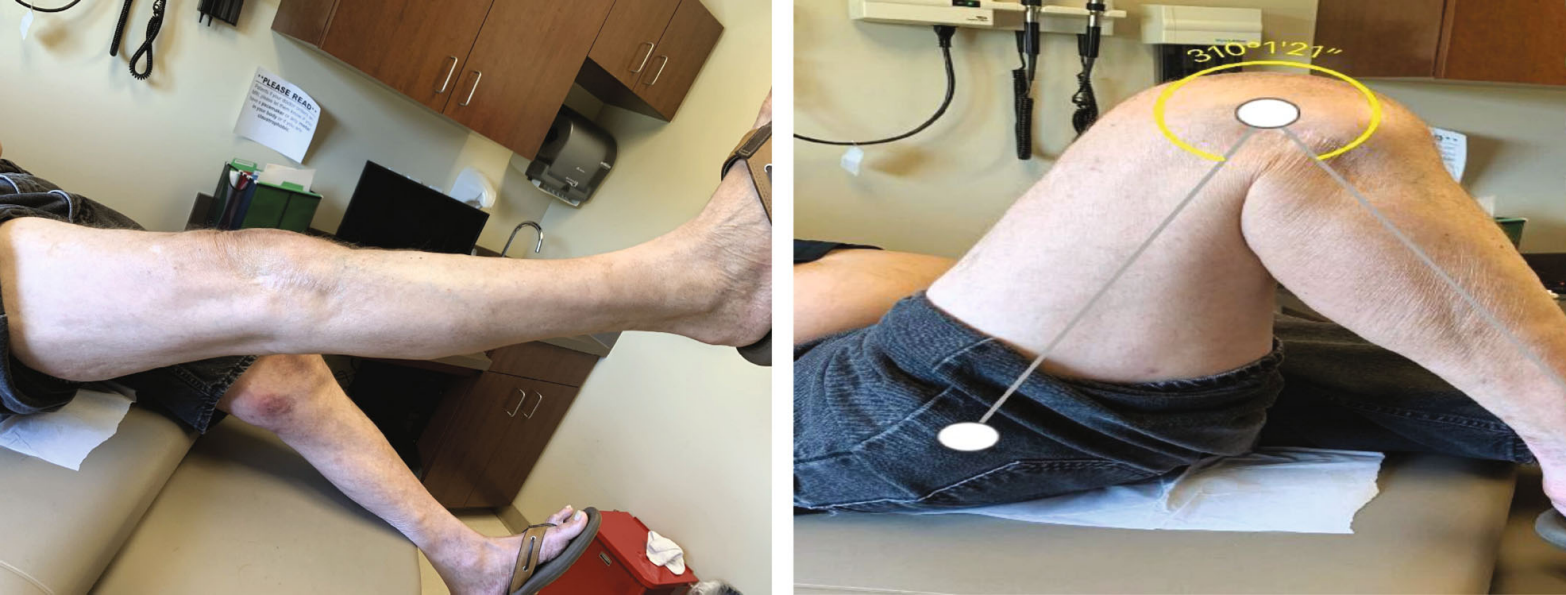
Office photos of 0-130° extension and flexion at a six-month follow-up.

At his six-month follow-up appointment, the patient reported no pain with RATKA replacement. Postop X-rays demonstrate excellent alignment of the mechanical axis (Figure 4). The final range of motion is 0-130° flexion with stable varus and valgus stress testing (Figures 5 and 6).

## 4. Discussion

Robotics involved in various surgical procedures over the years has proven that it promotes a positive impact on patient care by enhancing inpatient recovery and expediting the time to discharge [4]. Involving technology in healthcare gives the user an advantage, which can yield positive results that may lead to a decrease in hospital costs while increasing healthcare management efficiency. Song et al. reported that the robotic arm-assisted total knee arthroplasty (RATKA) system improved the accuracy of implant positioning, decreased postoperative bleeding, and minimizes the amount of bone being removed when compared to the conventional jig-based TKA (JTKA) [5].

Conventional JTKA techniques use a blueprint for the implant positioning that prioritizes the patient's anatomical axis. Patients with longstanding osteoarthritis inclusive of osteology changes may have some extensive osteophyte formation with or without angular deformities that can lead to distortion of the kinematics within the native joint, thus altering the anatomical axis. The use of the robotic arm-assisted device to position the implant during arthroplasty takes into account both the anatomical and mechanical axes, and this technology gives the user an advantage when treating altered native joints. However, the conventional JTKA method may not yield the best estimate of implant positioning or bone resurfacing when such anatomical axis alterations have occurred. Sodhi et al. concluded that utilizing preoperative CT images to develop a plan for the RATKA allows appropriate assessment of the deformity preoperatively and execution of a plan for balanced and aligned total knee arthroplasty [8]. The ability to combine both the anatomical and mechanical axes in the preoperative planning and the use of intraoperative feedback on the kinematics of the joint from the robotic arm-assisted device help the surgeon with balancing and positioning the implant. The longevity of the implant depends on its position within the joint, and the balance of the joint takes into account the amount of flexion and extension, corrected angular deformities, and preservation of the delicate tissues that support the intra-articular capsule during movement [5].

Extra-articular deformities can be very challenging to correct and most often occur after a malunited fracture, and femoral malunion deformities can result in distal femoral recurvatum with varus deformity leading to severe osteoarthritis [7]. Prior case reports and publications using the RATKA technique have reported success with the robotic arm-assisted devices in total knee arthroplasty for angular deformities and extra-articular deformities of either the femur or tibia. The purpose of this case report is to present complex extra-articular deformities in both the femur and tibia that were corrected with a RATKA replacement. This patient had deformities of both of his femur and tibia, which altered his anatomical axis and changed the kinematics of his native joint thus changing his mechanical axis. Due to his previous malunion of his femur, this type of deformity predisposes the knee joint to recurvatum with varus deformity [3]. Rhee et al. demonstrated that nonadequately corrected bone deformities can lead to early failure of the total knee arthroplasty procedure, and this can disrupt the half-life of the implant [9]. Not only would the conventional JTKA method have been very difficult due to distal femur deformity but it also would not have taken into account the kinematic alterations of the native joint and would have prioritized the anatomical axis, thus predisposing this joint to hyperextension which may have resulted in a recurvatum knee and possibly early implant failure. Because of the use of the robotic technology, we were able to position the implant in appropriate alignment resulting in the correction of recurvatum as well as reestablishment of the mechanical axis and properly balanced knee. This was reflected in the final outcome with excellent range of motion, proper balance, and correction of deformity.

With further supporting data of RATKA's superior treatment when compared to the conventional JTKA in patients with bone deformities seeking surgical treatment by way of TKA, the discussion of RATKA replacements being superior to conventional JTKA replacements in the treatment of complex extra-articular deformities that alter the kinematics of the native joint and generate a mechanical axis may be warranted.
